# Genomic diversity of SARS-CoV-2 in Oxford during United Kingdom’s first national lockdown

**DOI:** 10.1038/s41598-021-01022-x

**Published:** 2021-11-02

**Authors:** Altar M. Munis, Monique Andersson, Alexander Mobbs, Stephen C. Hyde, Deborah R. Gill

**Affiliations:** 1 Gene Medicine Group, Nuffield Division of Clinical Laboratory Sciences, Radcliffe Department of Medicine, University of Oxford, Oxford, UK; 2 Oxford University Hospitals NHS Foundation Trust, Oxford, UK

**Keywords:** Epidemiology, Population screening, SARS-CoV-2

## Abstract

Epidemiological efforts to model the spread of SARS-CoV-2, the virus that causes COVID-19, are crucial to understanding and containing current and future outbreaks and to inform public health responses. Mutations that occur in viral genomes can alter virulence during outbreaks by increasing infection rates and helping the virus evade the host immune system. To understand the changes in viral genomic diversity and molecular epidemiology in Oxford during the first wave of infections in the United Kingdom, we analyzed 563 clinical SARS-CoV-2 samples via whole-genome sequencing using Nanopore MinION sequencing. Large-scale surveillance efforts during viral epidemics are likely to be confounded by the number of independent introductions of the viral strains into a region. To avoid such issues and better understand the selection-based changes occurring in the SARS-CoV-2 genome, we utilized local isolates collected during the UK’s first national lockdown whereby personal interactions, international and national travel were considerably restricted and controlled. We were able to track the short-term evolution of the virus, detect the emergence of several mutations of concern or interest, and capture the viral diversity of the region. Overall, these results demonstrate genomic pathogen surveillance efforts have considerable utility in controlling the local spread of the virus.

## Introduction

The coronavirus disease (COVID)-19 pandemic, caused by the novel severe acute respiratory syndrome coronavirus 2 (SARS-CoV-2), emerged in Wuhan, China, in late 2019^1–3^. To date there have been over 190 million confirmed COVID-19 cases and over 4 million deaths reported^4^. The United Kingdom (UK) was one of the regions with the largest COVID-19 epidemic during the first half of 2020. The number of SARS-CoV-2 positive cases rose sharply in March leading to the first national lockdown in the UK (23 March-15 June 2020), and by the end of June 2020, when the lockdown restrictions were starting to ease, there had been more than 40,000 COVID-19-related UK deaths^5^.

Understanding how new viruses evolve through transmission is crucial for crafting effective strategies to control infectious disease spread and to refine prevention approaches^6–8^. Early in the COVID-19 pandemic, SARS-CoV-2 likely faced limited evolutionary pressure due to its rapid spread combined with the lack of immunity worldwide^9^. The rate of viral mutagenic ability can alter virulence during outbreaks by increasing infection rates, helping viruses evade the host immune system, and creating drug resistance^10,11^. RNA viruses, such as influenza viruses, are usually characterized by their high mutation rates. In contrast, coronaviruses, including SARS-CoV-2, encode RNA polymerases that possess proofreading activity^12^ that help maintain the fidelity of RNA replication, thereby decreasing the mutation rate of the virus. Despite this, thousands of new SARS-CoV-2 variants have evolved since the beginning of the pandemic through host-to-host transmission, spontaneous nucleic acid damage, and recombination events^13^. Furthermore, recent studies have reported specific genotypes evolving through the mechanism of co-accumulation of mutations generating potentially more contagious and severe viral variants such as: B.1.1.7/alpha (UK), B.1.351/beta (South Africa), P.1/gamma (Brazil), and B.1.617/delta (India)^14^.

The first complete SARS-CoV-2 genome was published in January 2020^15^. Since then, there has been a considerable global effort to collect and share genomic data to inform key aspects of infectious disease control and pandemic response^7^. This tracking of viral epidemiology in real time has led to a clearer comprehension of COVID-19 epidemics globally^16–19^. In this retrospective study, we combine genetic and epidemiological data to investigate the genetic diversity of SARS-CoV-2 in Oxford, a typical UK city with a large (international) student population, during the first national lockdown of the UK. By focusing on viral isolates obtained at the John Radcliffe Hospital (the largest hospital in Oxford) between April and August 2020, we sought to investigate the short-term evolution of the virus via local transmission during a period with considerable restrictions on personal contact and travel. Through phylogenetic analyses, interpreted in the context of available epidemiological information, we aimed to understand local patterns of viral mutations in order to infer antigenic drift.

## Results

### Characteristics of SARS-CoV-2 identified from patient samples

**Figure 1:**
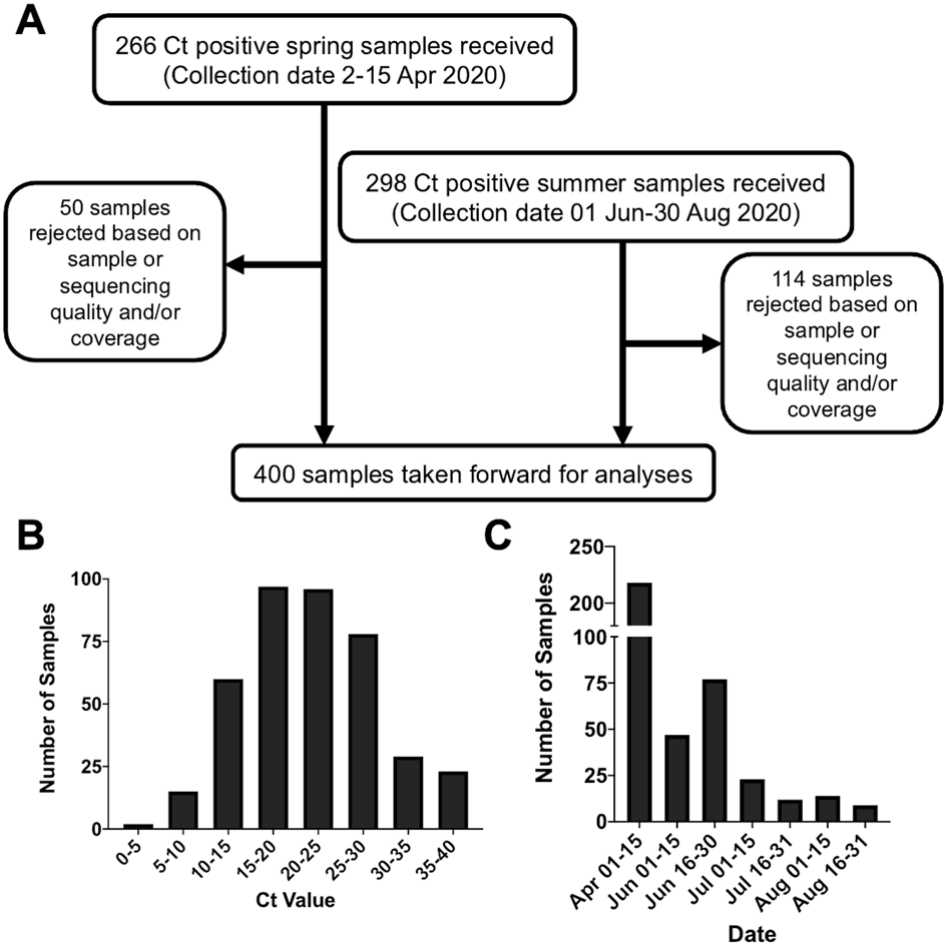
SARS-CoV-2 samples selected for genome sequencing. (**A**) Flowchart summarizing the timeline for sample receipt and subsequent sample selection process. Distribution of Ct (**B**) and collection date (**C**) for samples taken forward for analyses.

To identify the genetic variants of SARS-CoV-2 present in Oxford throughout the first UK national lockdown, a total of 563 samples were obtained, based on availability of residual RNA following diagnostic PCR testing. Samples were sequenced in two batches determined by their collection date (266 collected between April 2nd and 15th, hereafter referred to as the ‘spring samples’; 298 collected between 1st June and 30th August 2020, hereafter referred to as ‘summer samples’). Of the 536 samples, 400 yielded sequencing data of sufficient quality and were taken forward for analyses (Fig. 1A,C). We performed multiplexed, pooled, amplicon sequencing as described by the ARTIC network^20^ on Oxford Nanopore MinION Mk1B devices. Sequenced samples ranged in cycle threshold (Ct) from 3 to 38 (Fig. 1B)*.* Consistent with previous reports using the Oxford Nanopore platform, the coverage and the quality of the sequencing results correlated (Pearson *r* =  − 0.6632) with lower Ct values (Fig. S1)^21,22^. Incomplete genomes and low-quality sequences were primarily due to suboptimal RNA quality (e.g., low amplicon PCR yields) or amplicon dropout^23,24^. Despite the ambiguities, however, the resulting sequences could be used for variant calling or phylogenetic clustering in most cases (data not shown).

**Figure 2:**
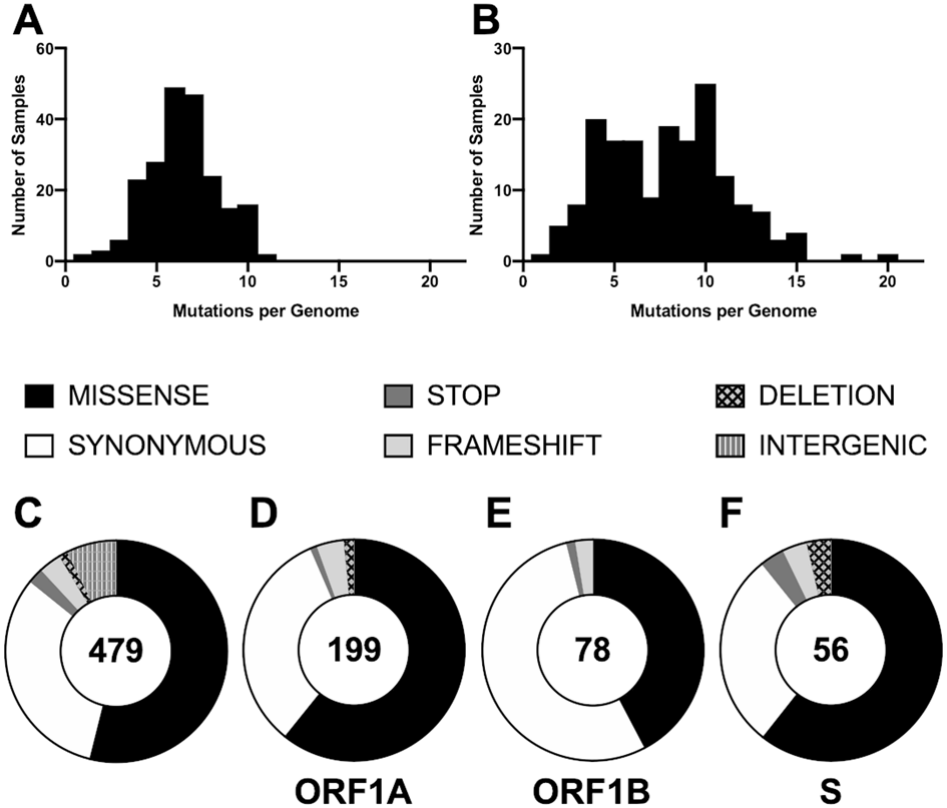
Summary of the distribution of single nucleotide polymorphisms detected. Histograms depicting the number of single nucleotide polymorphisms (SNPs) per viral genome detected in (**A**) spring and (**B**) summer samples. Pie charts illustrating the categorization, based on the type of mutation, of SNPs observed in all samples (**C**) in total or in open reading frames (**D**) ORF1A, (**E**) ORF1B, and (**F**) S, specifically. The values shown indicate the total number of unique SNPs observed.

From the 400 accepted samples we were able to detect a total of 337 unique variants (Table 1*).* For spring samples, these variants contained on average 6.4 (range 1–11) single nucleotide mutations compared with the Wuhan-Hu-1 reference genome (accession: MN9089047.3). This number rose to 7.8 (range 1–20) in the summer sample pool (Fig. 2A,B). Single nucleotide mutations were observed in all open reading frames of SARS-CoV-2 (Fig. 2D–F, and Fig. S2) with more than 50% comprising missense mutations (Fig. 2C). In addition, approximately one third of the single nucleotide variants were synonymous, such that they did not affect the amino acid sequence of the viral proteins—close to the proportion expected if the mutations were accumulating randomly. Approximately 7% of the mutations were in the intergenic, non-coding regions of SARS-CoV-2, while the majority of the mutations detected were in the genes coding for ORF1A, ORF1B, and S (Fig. 2D–F); when normalized to gene size, the mutation rates per gene were overall similar (Fig. S2, Supplementary Table 1).

**Table 1:** Summary of the total number of unique viral genomes detected in spring and summer samples.

	Samples sequenced	Number of unique viral genomes identified	Number of genomes detected in a single sample	Number of genomes detected in multiple samples
Spring	216	155	127	28
Summer	184	160	145	15
Total	400	337	294	43

### Genetic shifts can be observed between spring and summer samples

**Figure 3:**
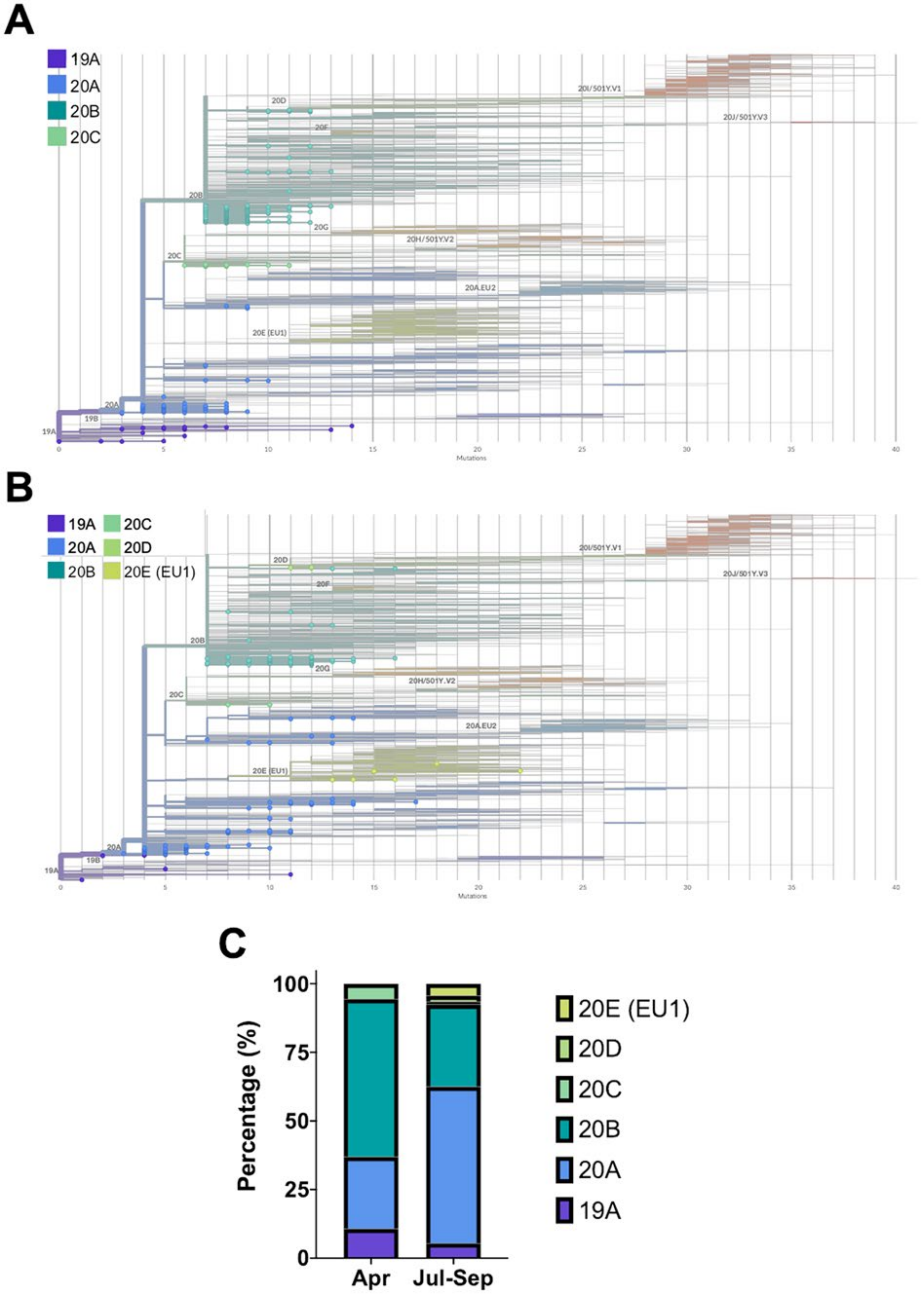
Phylogenetic analysis of SARS-CoV-2 genome sequences generated with respect to globally representative reference data sets. Smith-Waterman phylogenetic alignment of (**A**) spring and (**B**) summer samples on globally representative reference data sets (obtained from Nextstrain, sampled from GISAID^25–27^). The circles represent the viral genomes found in respective sample pools. The clades to which the specimens belong are indicated in the legends while global virus lineages identified to date are indicated on the tree. The phylogenetic tree was generated using Nextclade (version 0.14.2)^27^. (**C**) Bar graph highlighting the breakdown of viral lineages observed in spring and summer samples.

To better understand and visualize the genetic changes that occurred in the viral genome throughout the lockdown period, we performed phylogenetic analyses using the sequences we generated together with a globally representative reference dataset (obtained from Nextstrain, sampled from GISAID^25–27^) (Fig. 3A–C)*.* We observed that the spring samples clustered with clades 19A, 20A, 20B, and 20C. Of these, the 20A and 20B variants, the globally distributed base pandemic lines^27,28^, constituted the vast majority. In contrast, for the summer samples, while the majority of the variants also belonged to clades 20A and 20B, we were able to detect variants from newer clades, notably 20D and 20E (EU1). Although comprising less than 10% of the summer variants, clades 20D (concentrated in South America, southern Europe, and South Africa) and 20E (EU1) (concentrated in Europe), post summer 2020, were clustered together with the newer clades containing the B.1.1.7/alpha/20I (UK) and P.1/gamma/20 J (Brazil), and B.1.351/beta/20H (South Africa) variants respectively. These indicate the evolution and emergence of local viral lineages that gave rise to several variants of concern.

**Figure 4:**
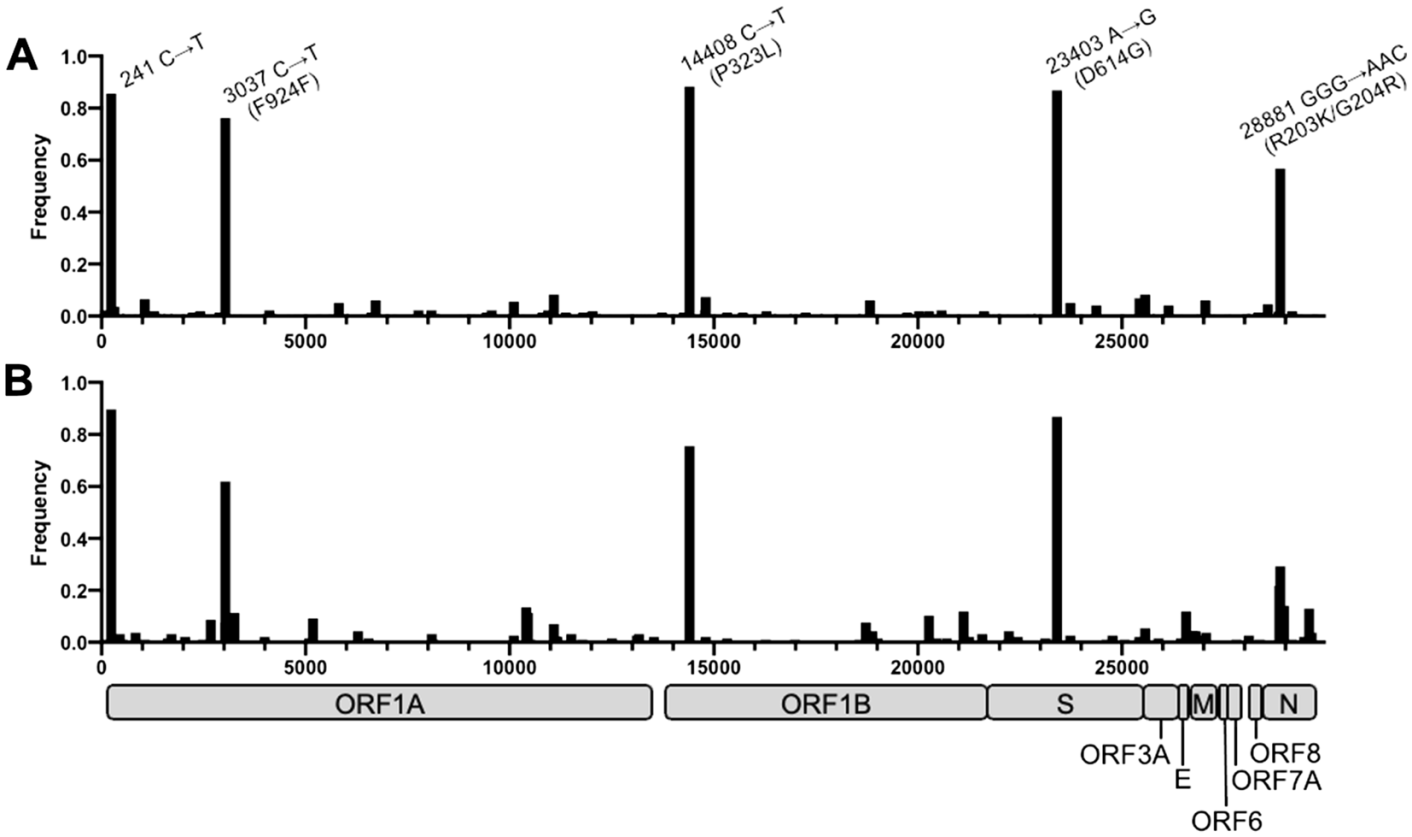
The frequency of SNPs detected at each genomic position, compared with reference genome MN908947.3. The frequency of viral genomes in (**A**) spring and (**B**) summer sample pools with a variant at each genomic position shown. The x-axes represent the length of the SARS-CoV-2 genome (to ~ 30,000 bp) with the genomic structure indicated underneath (in gray). The SNPs detected at five hotspots for both sample pools are labelled.

We observed that the overall number and distribution of unique single-nucleotide polymorphisms (SNPs) in the viral genome increased between the spring and summer samples from 243 to 283 (Fig. 4A,B, and in log-scale, Fig. S3). However, in both sample pools, we detected five shared major SNPs: 241C → T (intergenic), 3037C → T (F924F, ORF1A), 14408C → T (P323L, NSP12 in ORF1B), 23403A → G (D614G, spike protein), and 28881GGG → AAC (R203K/G204R, nucleocapsid protein). The widely examined D614G mutation in the viral spike protein emerged early and the frequency of variants containing this mutation increased rapidly worldwide in March–April 2020^29^. As a result, the D614G mutation now dominates the global pandemic and, in addition, this mutation is key in differentiating clades 19 and 20^28^. It has been reported that viral particles containing the D614G spike mutation replicate at an increased rate in primary human airway tissues and demonstrate enhanced viral fitness^30,31^, supporting the rapid spread of this mutation. A recent population genetic and phylodynamic study of more than 25,000 sequences in the UK showed that, following its introduction into the population, the D614G variant went through an exponential growth in infection rate consistent with a selective advantage (i.e., positive selection due to viral fitness) over the original 614D variant^32^. Moreover, researchers reported that the samples containing the variant were also associated with higher viral loads. Here, approximately 87% of all samples that were sequenced contained this mutation (Fig. 4A,B).

The 14408C → T SNP (Fig. 4) is also located in a protein essential for viral replication, specifically, the viral RNA-dependent RNA polymerase (RdRp)^33^; hence it has the potential to alter replication machinery and compromise the fidelity of the RNA replication. While this amino-acid altering SNP was observed to be common in early European isolates (first detected in Italy in February 2020,^34^) its potential effects on SARS-CoV-2 replication, infectivity, and fitness are still to be fully understood. In contrast to the other four major SNPs, we observed that the frequency of the 28881GGG → AAC mutation was considerably decreased (57 to 29%) in the summer samples compared with the earlier spring samples; mainly found in the European variants, the R203K/G240R affects the serine-arginine rich motif of the viral nucleoprotein^34^. It is postulated that the resulting mutations interfere with the phosphorylation of the serine residues in the motif thus modulating the normal function of the protein. This mutation has also been of particular interest as analogous modifications in SARS-CoV nucleocapsid have previously been linked to reduced viral pathogenicity^35^. We hypothesize this may be the reason behind de-selection of R203K/G240R out of the population and its reduced frequency in the summer samples.

### Evaluation of spike protein genetic diversity in oxford samples

**Figure 5:**
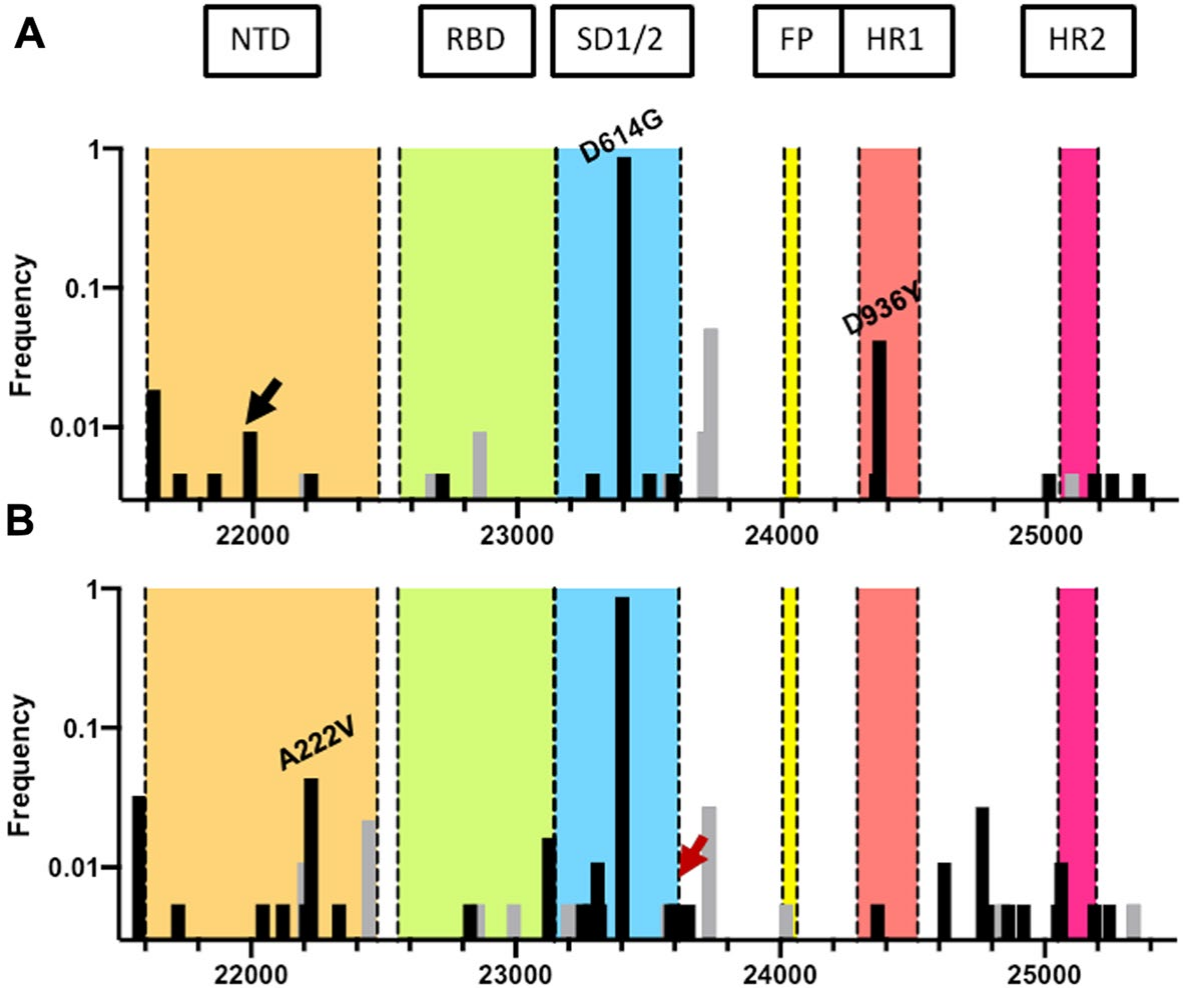
The frequency of SNPs detected in the S protein open reading frame. The frequency of viral genomes in (**A**) spring and (**B**) summer sample pools with a variant in the S protein open reading frame. Several key mutations are labelled with synonymous mutations indicated in light gray. The black and red arrows indicate the ∆V143/Y144 and P681H. The major domains of the S protein are indicated in different colors. *NTD* N-terminal domain (orange); *RBD* receptor binding domain (green); SD1/2: subdomains 1 and 2 (blue); *FP* fusion peptide (yellow); *HR1* heptad repeat 1 (red); *HR2* heptad repeat 2 (magenta).

The SARS-CoV-2 spike (S) glycoprotein mediates virus entry into cells via interactions with the human angiotensin-converting enzyme 2 (ACE2)^32,36^ as well as other factors^37,38^. Due to its indispensable function in viral infection, S protein is a major target for antibodies during immunological responses. It comprises six major domains: N-terminal domain, receptor binding domain (RBD), subdomains 1 and 2, fusion peptide, and heptad repeats 1 and 2^39^. Mutations of key residues throughout the S protein, specifically in the RBD, may play important roles in enhancing interactions with its receptor and thereby increasing infectivity of the virus. In contrast, other mutations might result in antigenic drift and escape making the virus less amenable to neutralization by antibodies. As noted above, one of the most significant S protein mutations is the D614G SNP in subdomain 1. Experimental studies have demonstrated that the mutation increased the infectivity of the virus by altering the receptor binding confirmation, thereby increasing the fusogenicity^29,31,40^. Unsurprisingly, we observed the D614G mutation in more than 80% of the samples we sequenced (Fig. 5A,B).

Approximately, 71% of all SNPs we detected (n = 39) encoded non-synonymous mutations in the S protein (Table 2). Several of the identified mutations were of significance. Mutation D936Y, that was particularly widespread in Sweden in spring 2020, was also detected in approximately 5% of the Oxford spring samples. Located in the heptad repeat 1 domain of the viral fusion core, the D936 residue is thought to play an important role in the post-fusion assembly of the glycoprotein. 3D modelling analyses have demonstrated that the D936Y mutation may destabilize the post-fusion structure of S resulting in reduced infectivity^41^; potentially explaining why this mutation was found at lower frequency in the later summer sample population. In contrast, the A222V mutation was first detected in the period from July to November 2020^42^. Especially affecting the UK, Spain, and Italy, A222V variants on a D614G background are regarded as the first diverging viral mutants of the 20E (EU1) clade^43^. In line with these findings, we observed the emergence of the A222V SNP in the summer samples while being absent in the earlier spring pool. In September 2020, a new variant emerged in south-east England termed B.1.1.7 (now known as the alpha variant)^44,45^. Amongst a total of 17 non-synonymous mutations compared with the Wuhan-Hu-1 variant, the eight mutations located in the spike protein (∆H69/V70, ∆Y144, N501Y, A570D, P681H, T716I, S982A, and D1118H) are thought to confer improved infectivity and transmissibility through enhanced immune evasion and increased binding affinity for ACE2^46,47^. Strikingly, two of the mutations, namely ∆V143/Y144 and P681H, can be detected in Oxford samples as early as April 2020 highlighting the slow evolution and emergence of the B.1.1.7/alpha lineage in England.

**Table 2:** The list of all non-synonymous mutations identified in the S protein open reading frame and the count of viral genomes containing the variants in each sample pool.

Mutation	Number of samples	
	Spring	Summer
L5F	–	6
R21I	4	–
L54F	1	1
S98F	1	–
ΔV143/Y144	2	–
S161Y	–	1
*221	–	1
L216F	–	1
F220I	1	–
A222V	–	8
G257D	–	1
T385I	1	–
*423	–	1
A522S	–	3
*607	–	1
D574Y	–	1
A575S	1	–
E583Q	–	2
D586Y	–	1
D614G	187	160
R646L	1	–
Q675H	1	1
P681H	–	1
*698	–	1
G932C	1	–
D936Y	9	1
A1020V	–	2
V1068F	–	5
ΔT1076	–	1
H1101Y	–	1
T1120I	–	1
E1150D	1	–
D1163Y	–	1
G1167V	–	2
E1207D	1	–
Y1209F	–	1
V1228L	–	1
M1229I	1	–
P1263L	1	–

## Discussion

During viral outbreaks, the identification and tracking of genetic variants can play a significant role in orienting the public health approaches used to control the spread of the SARS-CoV-2 virus and to develop therapies against it^48–50^. Current next-generation sequencing technologies, which offer ultra-high throughput, scalable, and fast parallel sequencing techniques, provide researchers with a unique opportunity to understand the spread of pathogens and track their genomic evolution in real-time. Furthermore, incorporation of the Nanopore sequencing system to such genomic surveillance efforts expands the potential reach of the studies owing to the superior accessibility and affordability of the sequencing platform.

Before the first UK national lockdown in March 2020, high volumes of national and international travel led to the establishment and co-circulation of more than a thousand identifiable SARS-CoV-2 lineages contributing to the spread of the COVID-19 epidemic^13^. While the lockdown was successful in controlling and decreasing the epidemic reproduction number, it also facilitated the survival of widespread lineages eliminating most local variants with low prevalence. Transmission of remaining variants at a local level shaped the later waves of epidemics in the UK driven by the advantageous characteristics conferred on lineages via specific mutations in the viral genome.

In this study, we performed detailed genetic analysis of viral strains circulating in Oxford in the first half of 2020. A total of 479 genetic variants were detected in Oxford, with 60.8% involving changes in the amino acid sequence compared with the Wuhan-Hu-1 reference sequence. The five major SNPs identified in Oxford samples have been previously identified and investigated in recently published genomic surveillance studies^29,33,34^. Three of the five SNPs were missense mutations in protein coding regions of the viral genome. D614G was the most prominent SNP detected, located upstream of the S1 cleavage domain of the spike glycoprotein^29^, and has been of great interest during the early phases of the pandemic. The other two SNPs, P323L and R203K/G204R, are located in the RdRp and nucleocapsid, respectively. Interestingly, we observed that the R203K/G204R SNP was decreased in the summer samples compared with its frequency in the spring samples, possibly being de-selected due to reduced viral pathogenicity^35^. Overall, we observed a considerable increase in the number of SNPs throughout all coding sequences of the virus in the summer samples (Fig. 2 and Fig. S4). This was also mirrored in the number of SNPs identified, which rose from 6.4 to 7.8 per genome on average (Fig. 2). We postulate that the overall increase in variants observed is representative of the adaptation of the virus to specific genetic backgrounds encountered in the host (e.g. in modulating the antiviral immune response) as well as adaptation to other unknown factors in the region.

The variety of different clades in the spring samples (Fig. 3) suggests multiple unrelated introductions of unique viral strains into Oxford prior to lockdown, possibly due to foreign travel and visitors from the wider geographic area. However, we speculate that the shift in clades observed in samples collected after the lockdown period are more likely the result of viral adaptation and evolution within the local population. The latter is based on local population genetic backgrounds and other unknown evolutionary pressures due to limited interaction with the wider UK population and restricted overseas travel. Furthermore, the diversity of lineages reported in this study indicate that different SARS-CoV-2 strains with varying mutation patterns co-exist in the Oxford population. Variants detected early in the pandemic were not lost from, and are still evident in, the summer samples. Considering the population size of Oxford (152,450^51^), the number of samples analyzed in this study is relatively small (~ 0.026%). Although the samples came from a geographically strict region, at a time of limited local and international travel and when personal contact was significantly restricted, under-sampling of genomes makes it hard to acquire a precise picture of viral transmission. Furthermore, all samples were obtained from Oxford University Hospitals laboratories, which may not be representative of asymptomatic transmission in the general population. In addition, the phylogenetic results obtained during the early phases of the pandemic should be interpreted carefully as the number of mutations required for defining clades/lineages are often small (e.g., D614G is the only mutation differentiating clades 19 and 20A).

In order to sustain an effective public health response, genomic surveillance studies should be undertaken for an extended period of time. While nationwide or global efforts are likely to be confounded by the number of independent introductions of the viral strains into a region, ‘closed system’ viral tracking studies, such as the one described here, have an untapped potential to inform clinical interventions. The combination of the genomic phylodynamic information from these studies with clinical data (e.g., age, sex, disease phenotype, hospitalization and vaccination history) can demonstrate changes in transmissibility and pathogenicity of new variants of concern (VOCs) (e.g. Indian/delta variants). This can also inform localized outbreaks, collecting essential data on the impact on SARS-CoV-2 evolution on individual immunity (from vaccines or natural infection) and the effectiveness of diagnostics and other therapeutic interventions. The properties of the closed system surveillance study will allow for precise quantification of the emergence, geographic spread, and reintroduction of specific VOCs, while also supporting classical surveillance strategies by evaluating the evolutionary drivers (e.g. type of therapeutic intervention or vaccine) and estimating the transmission levels in a controlled population. While similar closed system genomic surveillance have not been reported to date, early in the pandemic, several studies were able to illustrate the utility of this approach demonstrating correlations of SARS-CoV-2 infection spread in the US with interstate travelling patterns of individuals^9,52^. In addition, a study conducted in the East of England over a 5-week time period utilized genomic surveillance data to refute linked transmission between patients and health-care workers as a mechanism to monitor and target infection control measures^22^. In a similar study conducted in Italy, Giovanetti and colleagues were able to ascertain the dynamic shifts in SARS-CoV-2 transmission in response to the public health interventions by combining viral genetic and epidemiological data analyses^53^. Lastly, by coupling genetic variant data with mathematical modelling approaches researchers are able to pinpoint phylogenetic relationships between subpopulations more precisely, as well as identify sporadic clusters acting as hidden viral reservoirs throughout the pandemic^54^.

Ongoing efforts to track viral genomes in this way will allow us to answer critical questions, not only about the evolution of SARS-CoV-2 but also the impact of control measures designed to limit its epidemic spread. Furthermore, the approach taken here complements the information available on rapidly expanding, public databases of SARS-CoV-2 sequences. Specifically, this approach focuses the collection of genomic data into settings in which extensive current and historic clinical information can be accessed, and utilized, to investigate fundamental questions about our evolving relationship with SARS-CoV-2.

## Methods

### Ethical considerations

All samples were collected as nose and/or throat swabs in viral transport media during routine clinical care and stored at − 80 °C. No specific consent record is taken, at Oxford University Hospitals NHS Foundation Trust (OUH), for the sampling of nose and/or throat swabs, so there is no opportunity for the consent for use of samples for any other purposes than the original virus screening, to be declined. Samples were provided to the sequencing lab, with no identifiers nor means of linking them back to the patients. The protocol for the use of surplus clinical samples at OUH John Radcliffe Hospital, as described in this manuscript, was reviewed by the Institutional Review Board of OUH and determined to constitute service evaluation and development. As such, this study was deemed to not require research ethics review.

### Nucleic acid extraction from clinical specimens

RNA was extracted using the QIAsymphony SP instrument with the DSP Virus/Pathogen Kit and the Complex200_OBL_V4_DSP protocol^55^. Aliquots of eluted RNA were immediately stored at − 80 °C. The samples were mostly restricted to hospitalized patients and symptomatic staff. 266 samples were collected between 2nd and 15th April 2020. 298 samples were collected between 1st June and 30th August 2020.

### Diagnostic qRT-PCR

Reference quantitative reverse-transcription polymerase chain reaction (qRT-PCR) assays were performed to determine sample cycle threshold (Ct) values, using either the Abbott RealTime or Altona RealStar SARS-CoV-2 detection assays according to manufacturers’ instructions.

### Genomic sequencing with ARTIC tiled amplicons

Whole genome amplification of SARS-CoV-2 genome was performed using the ARTIC network protocol with the V3 primer set^20^. cDNA was synthesized from previously extracted RNA samples. No sample dilution was performed to normalize samples by Ct value ranges. A one-step reverse transcription polymerase chain reaction (PCR) was performed using LunaScript RT SuperMix Kit (NEB, #E3010), followed by multiplexed PCR in two non-overlapping pools using Q5 DNA polymerase (NEB, #M0491). Amplicon pools were indexed using Oxford Nanopore Native barcoding reagent sets EXP-NBD104 and EXP-NBD114 or EXP-NBD196 for 24plex and 96plex sequencing runs. Indexed samples were pooled and amplicon DNA sequences were determined using an Oxford Nanopore MinION MK1B instrument with R9.4.1 flow cells. Base-calling was performed using Guppy version 3.1.5 for Windows with a Phred quality cut-off of > 9. Prepared libraries were sequenced for ∼24–48 h.

### Genome assembly and variant identification

Reference-based genome assembly was performed using the ARTIC network bioinformatics pipeline v1.3.0 for COVID-19 (https://github.com/artic-network/fieldbioinformatics). Briefly, base-called reads were de-multiplexed with Guppy v3.1.5. Reads were mapped to the SARS-CoV-2 reference (GenBank accession MN908947.3) using Minimap2 with Medaka used for error correction. Viral genome consensus sequences were determined and variants with quality score > 400 were accepted. A summary of all samples with associated collection dates, Ct values, and run-barcode information can be found in Supplementary Table 2.

### Phylogenetic analysis

Phylogenetic analysis was performed via Nextstrain’s online tool Nextclade (version 0.14.2). Sequenced SARS-CoV-2 genomes as well as globally representative reference dataset (December 2019-June 2021, obtained from Nextstrain, sampled from GISAID^25–27^) were clustered using Augur, the phylodynamic pipeline provided by Nextstrain, and visualized using Auspice.

## Supplementary Information

Supplementary Information 1.

Supplementary Information 2.

## Data Availability

Complete assemblies and all raw sequencing data were submitted to NCBI under Bioproject ID PRJNA749667.
