# Supplementary material for: Genomic diversity of SARS-CoV-2 in Oxford during United Kingdom’s first national lockdown
Source: Sci Rep. 2021 Nov 2;11:21484. doi: 10.1038/s41598-021-01022-x (PMC8564533; doi:10.1038/s41598-021-01022-x)
Supplement: Supplementary file 1 — Supplementary Information 1. [file 41598_2021_1022_MOESM1_ESM.pdf]

Genomic Diversity of SARS-CoV-2 in Oxford During United Kingdom's First National Lockdown

Altar M Munis[1], Monique Andersson[2], Alexander Mobbs[2], Stephen C Hyde[1], Deborah R Gill[1*]

[1]Gene Medicine Group, Nuffield Division of Clinical Laboratory Science, Radcliffe Department of Medicine, University of Oxford, Oxford, UK

[2] Oxford University Hospitals NHS Foundation Trust, Oxford, UK

[*]*Correspondence to deborah.gill@ndcls.ox.ac.uk.*

Supplementary Materials

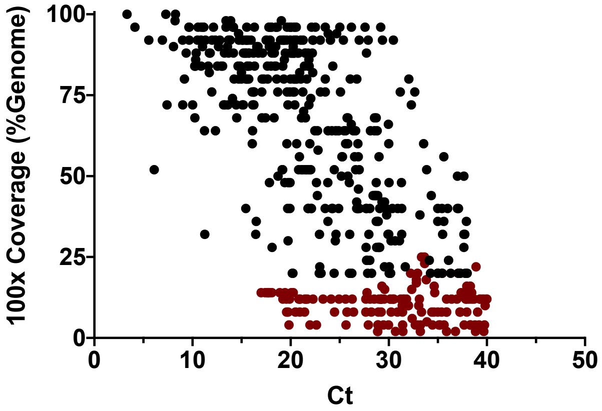

Figure S1: Plot of SARS-CoV-2 genome coverage (100X) against real-time reverse transcription Ct values for all samples sequenced in this study. The red data points indicate samples rejected based on sample or sequencing quality and/or coverage.

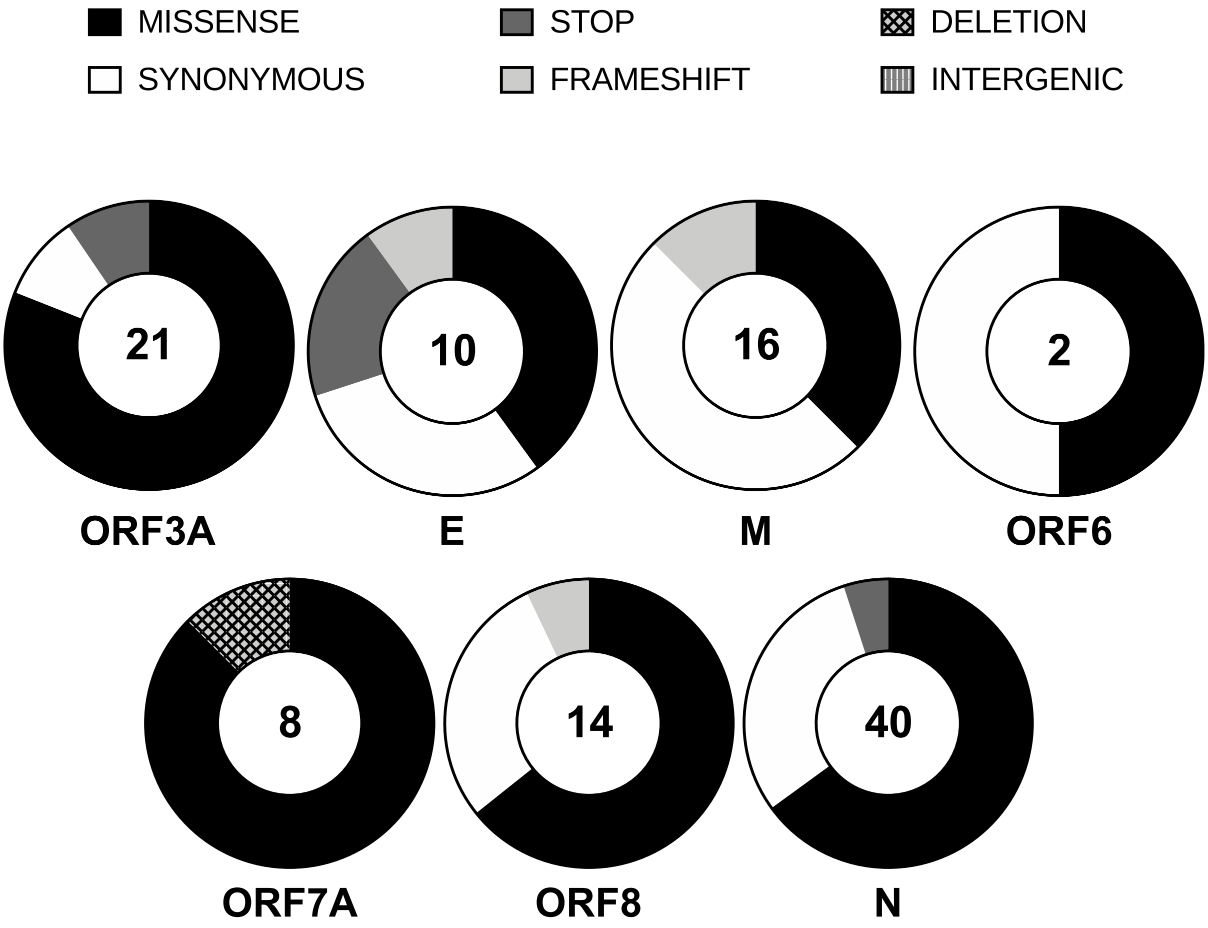

Figure S2: Pie charts illustrating the categorization of the type of mutation of SNPs observed in all samples in open reading frames ORF3A, E, M, ORF6, ORF7A, ORF8, and N. The values indicate the total number of unique SNPs observed.

Supplementary Table 1: The mutation rates normalized to gene size.

GENE	MISSENSE	SYNONYMOUS	STOP	FRAMESHIFT	DELETION	TOTAL	GENE SIZE (kb)	MUTATIONS/kb
ORF1A	121	65	2	8	3	199	13.218	15.1
ORF1B	33	42	1	2	0	78	7.788	10.0
S	34	16	2	2	2	56	3.849	14.5
ORF3A	17	2	2	0	0	21	0.828	25.4
E	4	3	2	1	0	10	0.228	43.9
M	6	8	0	2	0	16	0.669	23.9
ORF6	1	1	0	0	0	2	0.186	10.8
ORF7A	7	0	0	0	1	8	0.366	21.9
ORF8	9	4	0	1	0	14	0.366	38.3
**N**	26	12	2	0	0	40	1.26	31.7

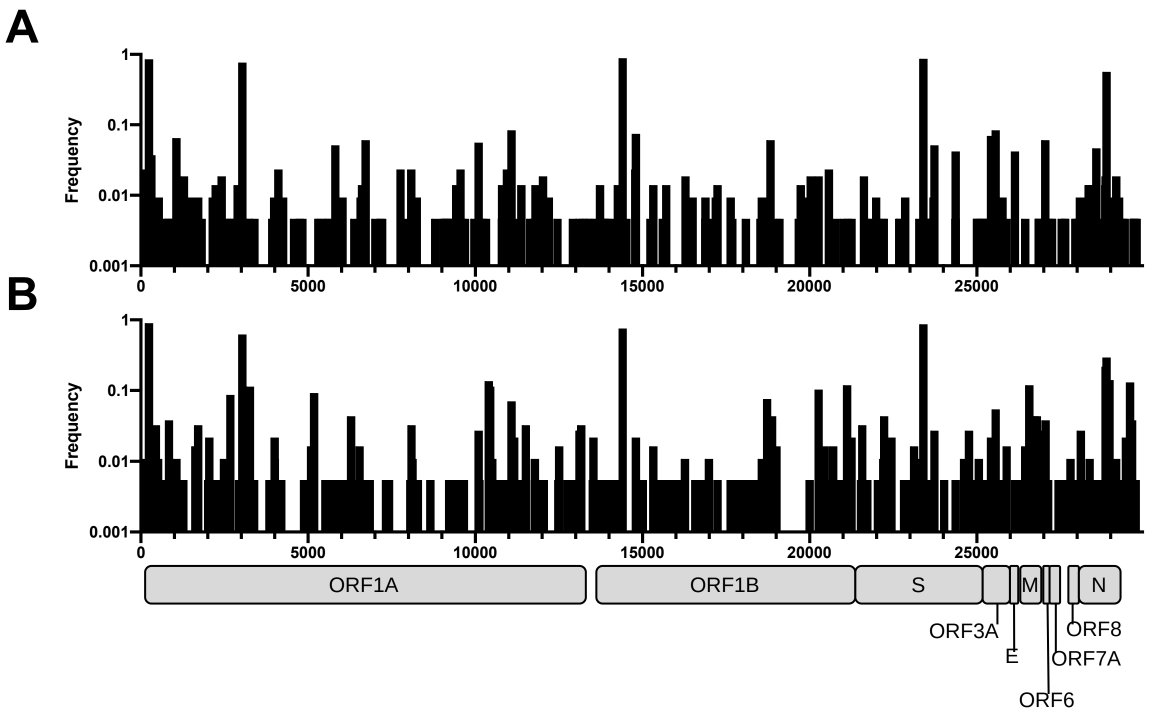

Figure S3: The frequency of SNPs detected, with respect to reference genome MN908947.3, at each genomic position. The frequency of viral genomes in **(A)** spring and **(B)** summer sample pools with a variant at each genomic position. The x-axes represent SARS-CoV-2 genome positions with SARS-CoV-2 genomic structure indicated underneath (in light gray).

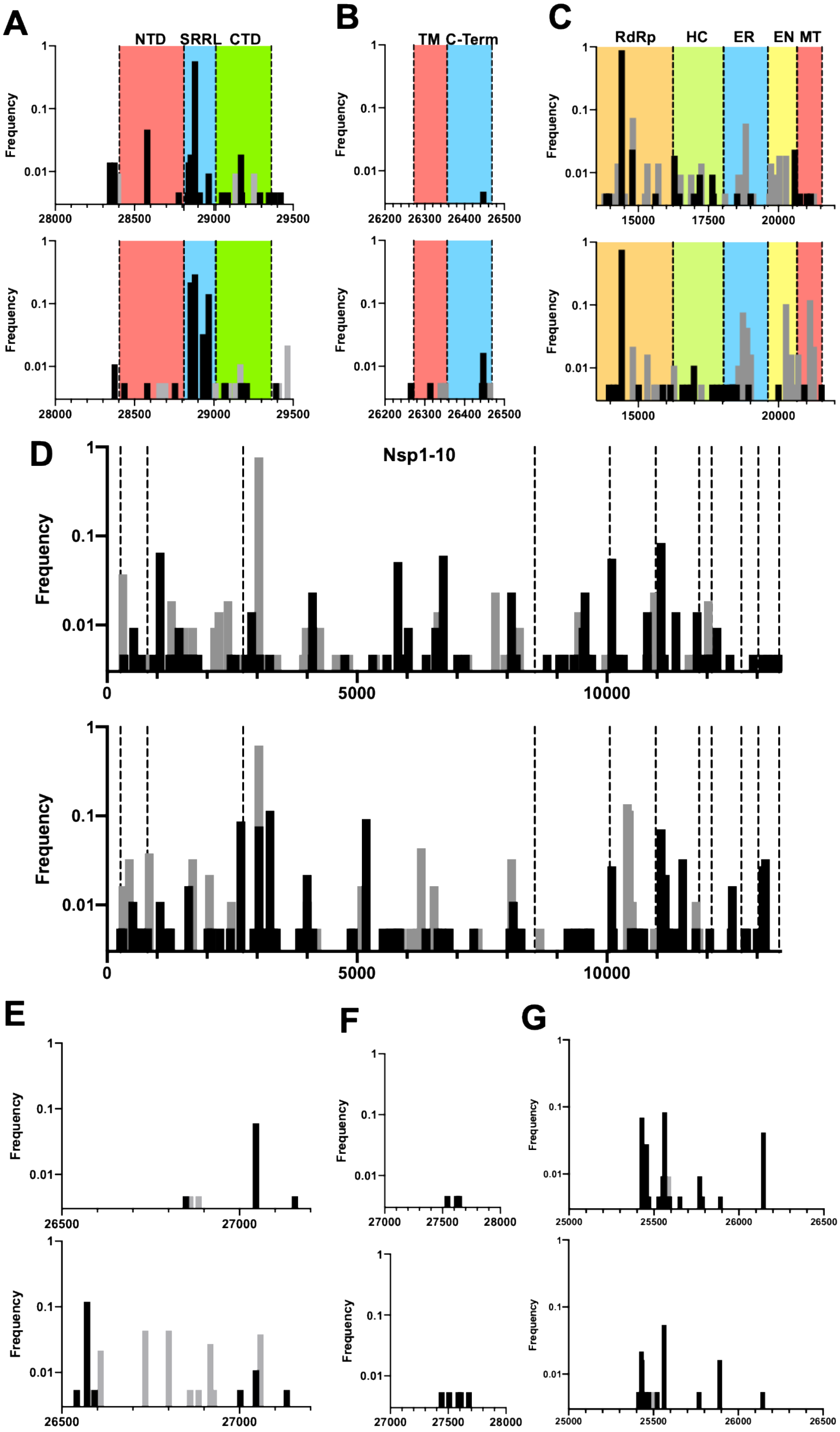

A
NTD
SRRL
CTD
B
TM
C-Term
C
RdRp
HC
ER
EN
MT
D
Nsp1-10
E
F
G
Frequency
1
0.1
0.01
28000
28500
29000
29500
26200
26300
26400
26500
15000
17500
20000
0
5000
10000
26500
27000
27500
28000
25000
25500
26000

Figure S4: The frequency of SNPs detected in SARS-COV-2 open reading frames **(A)** N, **(B)** E, **(C)** ORF1B, **(D)** ORF1A, **(E)** M, **(F)** E, and **(G)** ORF3A. Major domains of several key open reading frames are color-coded and indicated with vertical dotted lines. NTD: N-terminal domain; SRRL: serine-arginine (SR)-rich linker domain; CTD: C-terminal domain; TM: transmembrane domain; C-Term: C-terminal; RdRp: RNA-dependent RNA polymerase; HC: helicase; ER: endoRNAse; EN: exonuclease; MT: methyltransferase. Synonymous mutations are indicated in light gray.
